# Structural Analysis of Glycine Sarcosine N-methyltransferase from *Methanohalophilus portucalensis* Reveals Mechanistic Insights into the Regulation of Methyltransferase Activity

**DOI:** 10.1038/srep38071

**Published:** 2016-12-09

**Authors:** Yi-Ru Lee, Te-Sheng Lin, Shu-Jung Lai, Mu-Sen Liu, Mei-Chin Lai, Nei-Li Chan

**Affiliations:** 1 Institute of Biochemistry and Molecular Biology, College of Medicine, National Taiwan University, Taipei 100, Taiwan; 2 Department of Life Sciences, National Chung Hsing University, Taichung 402, Taiwan; 3 Institute of Biochemistry, National Chung Hsing University, Taichung 402, Taiwan

## Abstract

Methyltransferases play crucial roles in many cellular processes, and various regulatory mechanisms have evolved to control their activities. For methyltransferases involved in biosynthetic pathways, regulation via feedback inhibition is a commonly employed strategy to prevent excessive accumulation of the pathways’ end products. To date, no biosynthetic methyltransferases have been characterized by X-ray crystallography in complex with their corresponding end product. Here, we report the crystal structures of the glycine sarcosine N-methyltransferase from the halophilic archaeon *Methanohalophilus portucalensis (Mp*GSMT), which represents the first structural elucidation of the GSMT methyltransferase family. As the first enzyme in the biosynthetic pathway of the osmoprotectant betaine, *Mp*GSMT catalyzes N-methylation of glycine and sarcosine, and its activity is feedback-inhibited by the end product betaine. A structural analysis revealed that, despite the simultaneous presence of both substrate (sarcosine) and cofactor (S-adenosyl-L-homocysteine; SAH), the enzyme was likely crystallized in an inactive conformation, as additional structural changes are required to complete the active site assembly. Consistent with this interpretation, the bound SAH can be replaced by the methyl donor S-adenosyl-L-methionine without triggering the methylation reaction. Furthermore, the observed conformational state was found to harbor a betaine-binding site, suggesting that betaine may inhibit *Mp*GSMT activity by trapping the enzyme in an inactive form. This work implicates a structural basis by which feedback inhibition of biosynthetic methyltransferases may be achieved.

Methyltransferases participate in a wide variety of cellular processes, ranging from biosynthesis and conversion of cellular components to the regulation of gene expression and protein function^1^,^2^,^3^,^4^. Aberrant methyltransferase activities have been linked to significant biological consequences, and thus various regulatory strategies have evolved to properly control methyltransferases according to physiological needs^5^,^6^,^7^,^8^. Well known mechanisms include phosphorylation, oxidation, automethylation, ubiquitination and other post-translational events^9^,^10^,^11^,^12^,^13^. For the majority of methyltransferases that utilize S-adenosyl-L-methionine (SAM) as the methyl group-donating cofactor, accumulation of the demethylated cofactor S-adenosyl-L-homocysteine (SAH) may lead to product inhibition; SAH hydrolases relieve the inhibitory effect by breaking down SAH into adenosine and homocysteine^14^,^15^. Some methyltransferases involved in biosynthesis are subjected to feedback inhibition by the end product of their respective biosynthetic pathway. For example, phosphocholine, berberine, and mycophenolic acid shut down their own biosynthesis by acting as potent allosteric inhibitors of the enzymes phosphoethanolamine N-methyltransferase, norcoclaurine 6-O-methyltransferase, and demethylmycophenolic acid O-methyltransferase, respectively^16^,^17^,^18^. Alternatively, phosphatidylcholine achieves feedback inhibition by binding to membrane-attached phospholipid N-methyltransferase to induce its detachment from the membrane^19^. To date, no biosynthetic methyltransferase has been characterized by X-ray crystallography in the presence of its corresponding end product. In this study, we performed structural analysis on the glycine sarcosine N-methyltransferase (GSMT) from *Methanohalophilus portucalensis* strain FDF1^T^ (*Mp*GSMT) to understand how end product-mediated allosteric inhibition is achieved.

The survival of the halophilic methanoarchaeon *M. portucalensis* in high salinity environments requires *de novo* production of betaine, a common osmoprotectant whose intracellular accumulation protects cells against osmotic pressure^20^,^21^. Betaine biosynthesis is accomplished by three N-methylation steps in which glycine is sequentially converted to sarcosine, dimethylglycine and the final product betaine (Supplementary Fig. S1). Two dual-activity, SAM-dependent methyltransferases sharing an overlapping function are employed to carry out this three-step biosynthetic pathway as follows: *Mp*GSMT adds the first and second methyl groups to glycine, and the sarcosine dimethylglycine N-methyltransferase (*Mp*SDMT) catalyzes the second and third methylation events^20^,^22^. Similar to other commonly exploited regulatory strategies, the control of betaine production is achieved by betaine-mediated feedback inhibition of *Mp*GSMT, the first enzyme in the betaine biosynthetic pathway.

According to the NCBI Protein Database, *Mp*GSMT (GenBank: AEG64703.1) is annotated as a member of the Class I SAM-dependent methyltransferase superfamily and exhibits the highest homology with the vertebrate glycine N-methyltransferase (GNMT), a hepatic enzyme that helps maintain a proper cellular SAM:SAH ratio by consuming SAM and glycine to form SAH and sarcosine^23^,^24^. The pronounced sequence similarity between *Mp*GSMT and GNMT agrees with their overlapping activity as follows: both enzymes catalyze the SAM-dependent methylation of glycine to produce sarcosine^24^,^25^. Structural analysis revealed that, similarly to all Class I methyltransferases, the catalytic core of GNMT adopts the Rossmann fold, which bears the SAM and substrate binding pockets^26^,^27^,^28^. In the presence of SAM and the inhibitor acetate, GNMT assumes a closed conformation with the active site sealed from the bulk solvent by the N-terminal region and lid domain. The methyl transfer reaction can then occur via the S_N_2 mechanism^29^. Given the prominent functional and sequence similarity, it is expected that *Mp*GSMT should resemble GNMT in its overall structure and catalytic mechanism. However, there are outstanding distinctions between the two enzymes. First, while *Mp*GSMT is negatively regulated by the end product betaine^25^, the activity of GNMT is regulated by folate^14^,^30^. Second, both glycine and sarcosine can be methylated by *Mp*GSMT^25^, whereas GNMT only acts on glycine^31^,^32^. Here, we report structural characterization of *Mp*GSMT in a betaine-bound, catalytically inactivated state, which elucidates for the first time the structural basis by which the activity of a biosynthetic methyltransferase is regulated by end product-mediated feedback inhibition.

## Results

### The structure of *Mp*GSMT-sarcosine-SAH ternary complex in a catalytically inactivated state

**Figure 1:**
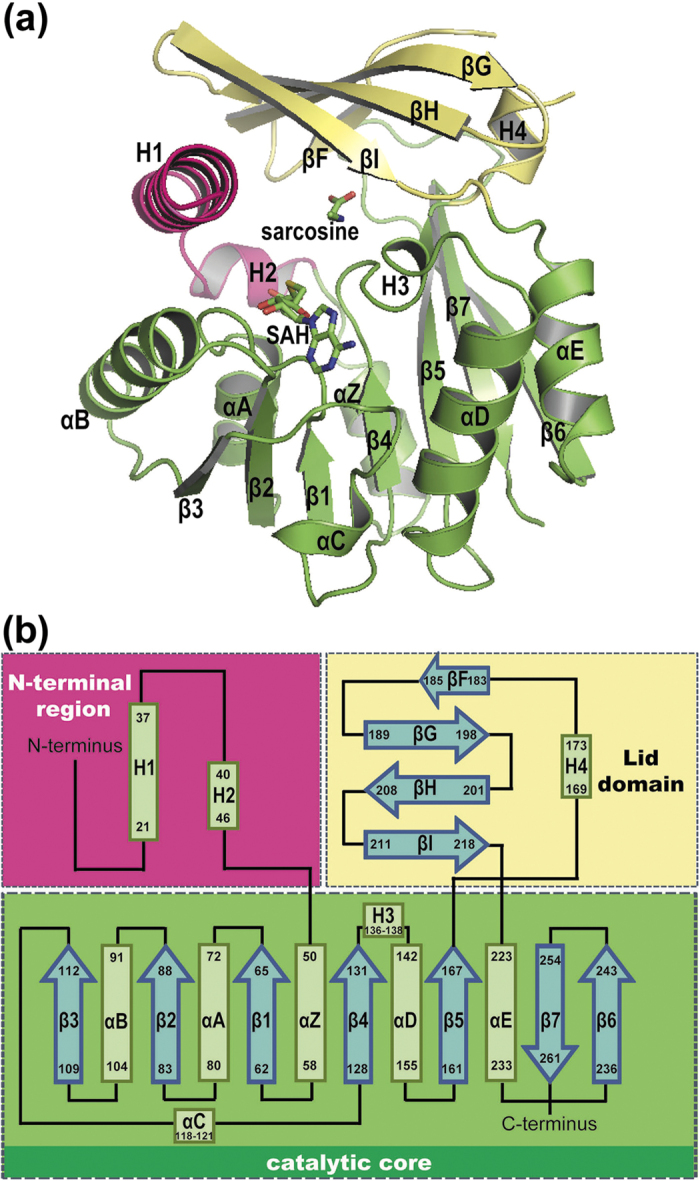
The structure of *Mp*GSMT in complex with sarcosine and SAH. (**a**) Cartoon showing the crystal structure of *Mp*GSMT in complex with sarcosine and SAH. The N-terminal region, catalytic core and lid domain are red, green and yellow, respectively; the bound SAH and sarcosine are shown as sticks. The secondary structural elements are labeled according to the scheme shown in panel b. (**b**) Topology diagrams of *Mp*GSMT with α-helices and β-strands shown as rectangles and arrows, respectively. The catalytic core of *Mp*GSMT is composed of seven β-strands flanked by seven helices, similar to other SAM-dependent methyltransferases^29^,^43^.

To understand how *Mp*GSMT is capable of catalyzing the N-methylation reaction with both glycine and sarcosine (N-monomethyl glycine), and how this enzyme is subjected to feedback inhibition by betaine, we first determined the crystal structure of *Mp*GSMT in complex with the sarcosine substrate and the SAH cofactor at 2.47 Å resolution (Table 1; Fig. 1a). Consistent with previous sequence-based prediction^26^,^27^, our structural analysis validated that *Mp*GSMT indeed belongs to the Class I methyltransferases, with a characteristic seven-stranded Rossmann-like α/β catalytic core responsible for substrate and cofactor binding (Fig. 1a). As expected, a structural comparison carried out using the DALI program showed that the overall structure of *Mp*GSMT most closely resembles vertebrate GNMTs (Z-scores over 20)^24^,^29^,^31^,^32^,^33^,^34^,^35^,^36^. The *Mp*GSMT and a rat GNMT structure^29^ can be superimposed with an RMSD of 2.1 Å over 230 structurally equivalent Cα atom pairs. Similar to GNMT, the catalytic core of *Mp*GSMT is further elaborated by a helical N-terminal region that precedes the β1 strand and a lid domain composed of a four-stranded antiparallel β sheet that inserts between the β5 strand and αE helix (Fig. 1).

**Figure 2:**
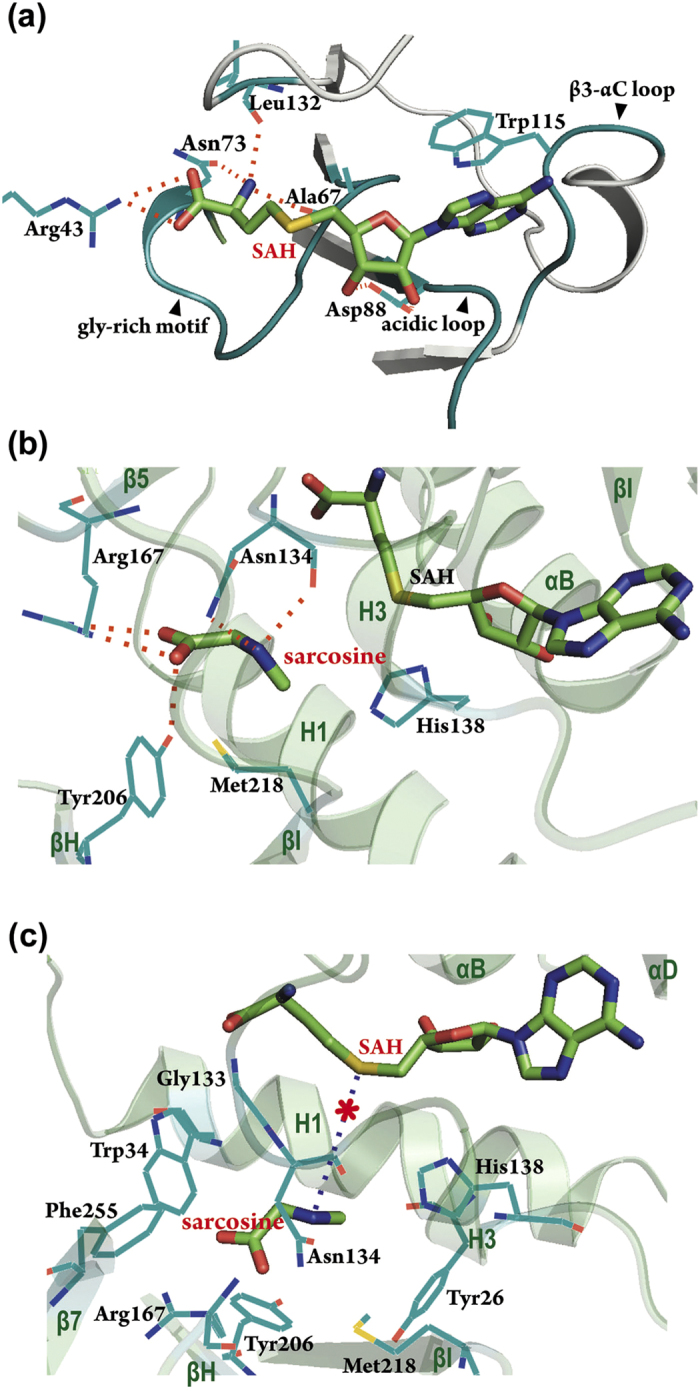
The structure of the *Mp*GSMT active site in a catalytically inactive state. (**a**) Structure of the SAH binding pocket. The cofactor-binding pocket is shaped by residues from the glycine-rich motif, the acidic loop and the loop region between β3 and αC. The guanidino group of Arg43 and the main-chain carbonyl group of Leu132 also contribute to cofactor binding by interacting with the carboxyl and amino groups of the cofactor’s methionine/homocysteine moiety, respectively. (**b**) Structure of the substrate-binding pocket. The bound substrate (sarcosine) is stabilized by a salt bridge and an H-bond between the carboxyl group and Arg167 and Tyr206, an H-bond between the amino group and Asn134, and van der Waals interactions with His138 and Met218. (**c**) Structure of the *Mp*GSMT active site. In this *Mp*GSMT-sarcosine-SAH structure, the methyl-accepting nitrogen atom and the cofactor’s sulfonium ion are 6.3 Å apart (blue dashed line), suggesting that the nitrogen is not yet in a “near attack” position to effectively initiate methyl transfer. The predicted location of the sulfur-attached methyl group is shown as a red asterisk. Salt bridges and H-bonds are shown as red dashed lines.

Clear electron density is visible for bound sarcosine and SAH (Supplementary Fig. S2), which allows both molecules to be precisely positioned in the active site of *Mp*GSMT. The cofactor-binding pocket mainly consists of residues from three universally present and conserved elements in Class I methyltransferases that interact with the three moieties of SAH (L-homocysteine, ribose, and adenine). These conserved elements are the glycine-rich motif (E/DXXXGXG; residues 65–73) and the short loop region between β3 and αC (residues 113–116), which interact with the L-homocysteine and adenine, respectively, and an acidic loop (residues 88–91) that contacts both the ribose and adenine (Fig. 2a). Residues are arranged to accommodate the shape of SAH and these three regions participate in ionic interactions, direct and water-bridged hydrogen bonds, van der Waals contacts and aromatic stacking to stabilize the bound cofactor. In addition, Arg43 from the N-terminal region and Leu132 from the β4-H3 loop also contribute to cofactor binding by anchoring the carboxyl and amino group of SAH, respectively. Notably, whereas structural analyses of other SAM-dependent methyltransferases have revealed the presence of a functionally significant interaction between an active site-located tyrosine and the positively charged sulfonium of the cofactor^7^,^29^,^37^,^38^,^39^,^40^,^41^, such an interaction was not observed in the *Mp*GSMT-sarcosine-SAH ternary complex. The functional relevance of this finding will be discussed below.

The substrate-binding pocket, where sarcosine or glycine can be recognized and positioned for N-methylation, involves residues from the lid and the core domains (Fig. 2b). The observed interactions between *Mp*GSMT and sarcosine indicate that both sarcosine and glycine can be specifically oriented by forming multiple contacts with the surrounding residues, including a salt bridge and a hydrogen bond formed, respectively, between the carboxyl group and the guanidino group of Arg167 and the phenolic hydroxyl of Tyr206; polar interactions between the amino group and the main-chain carbonyl and the side-chain amide of Asn134; and van der Waals contacts with Asn134, His138, and Met218. Among the residues involve in substrate binding, Arg167 is located at the C-terminal end of β5 and is spatially equivalent to the Arg169 of *A. halophytica* GSMT^42^, implicating a pivotal role for this residue in recognizing glycine and its N-methylated derivatives.

By adopting the ligand binding mode observed in the *Mp*GSMT-sarcosine-SAH structure, the amino group of sarcosine points directly at the sulfonium of the cofactor and approaches a position that appears suitable for engaging in the subsequent in-line attack on the S-methyl group of SAM (Fig. 2c). Intriguingly, in comparison with other SAM-dependent methyltransferases whose structures were determined in the presence of both the substrate and SAH (or the product and SAM)^29^,^43^, an unexpected distinctiveness was revealed in the *Mp*GSMT ternary complex structure. First, it is well documented that methyltransferases undergo extensive conformational changes upon substrate and cofactor binding. The apo forms of SAM-dependent methyltransferases frequently exhibit an open conformation in which the N-terminal region and the lid domain are distant from the core domain^36^,^44^,^45^,^46^. Hence, active site residues are predominantly solvent-exposed and ready to interact with the incoming substrate and cofactor. In contrast, the enzyme-substrate-cofactor ternary complexes tend to adopt a closed conformation in which the N-terminal region and the lid domain close upon the bound substrate and cofactor. This substrate/cofactor-induced conformational change not only places the methyl-accepting group and the donor methylsulfonium in proximity but also creates a narrow tunnel between the two reacting groups to facilitate methyl transfer. However, despite the concomitant presence of both sarcosine and SAH, the *Mp*GSMT active site remains partially open; the N-terminal H1 helix points away from the core domain without covering the substrate-binding pocket (Supplementary Fig. S3). Moreover, the distance between the methyl-accepting nitrogen of sarcosine and the cofactor’s sulfonium (the “N-S distance”) was 6.3 Å in the *Mp*GSMT structure (Fig. 2c), which is considerably longer than the N-S distances of 3.6–4.5 Å observed in other ternary complex structures^43^,^47^,^48^,^49^. Based on the observed partially open active site, the absence of a conserved polar/ionic interaction with the cofactor’s sulfonium, and the wide separation of the methyl-accepting nitrogen and sulfonium in our structure, we speculated that the structural state of *Mp*GSMT reported here may correspond to a functionally inactivated conformation of the enzyme.

To test the validity of our interpretation, we investigated the feasibility of preparing crystals of the *Mp*GSMT-sarcosine-SAM ternary complex. To this end, we first prepared crystals of the *Mp*GSMT-SAM binary complex (Table 1, Supplementary Fig. S2b) and transferred them to a substitute mother liquor that contained sarcosine and SAM. If the observed *Mp*GSMT structure does represent a catalytically inactivated state, we expected to observe the presence of both sarcosine and SAM in the active site without conversion to the products dimethylglycine and SAH. Indeed, X-ray diffraction analysis of the resulting crystals showed clear electron density maps corresponding to sarcosine and SAM in the active site (Table 1, Supplementary Fig. S2c). The B-factors of the atoms that constitute sarcosine and SAM are comparable to those of the surrounding protein residues, suggesting full occupancy for both ligands. Superimposition analysis revealed that the structures of *Mp*GSMT-sarcosine-SAH and *Mp*GSMT-sarcosine-SAM are highly isomorphous; no significant structural differences were observed, except for the minor reposition of sarcosine in response to the presence/absence of the sulfonium-linked methyl group (Supplementary Fig. S4). Our finding that crystals of the *Mp*GSMT-sarcosine-SAM ternary complex can be successfully prepared strongly indicates that the structure reported here represents a catalytically inactivated state of *Mp*GSMT.

To our knowledge, this is the first observation of a methyltransferase in its catalytically inactivated form despite the presence of both substrate and cofactor. Given that *Mp*GSMT is capable of switching between an inactivated and activated state depending on the ionic strength of the environment and the intracellular concentration of betaine^25^, we speculated that the observed *Mp*GSMT structure may, in fact, represent a functionally relevant, inactivated state of the enzyme that contains crucial structural information regarding the regulation of methyltransferase activity. The validity of this interpretation is addressed further in the following sections.

### Structural basis of betaine-mediated feedback inhibition of *Mp*GSMT

**Figure 3:**
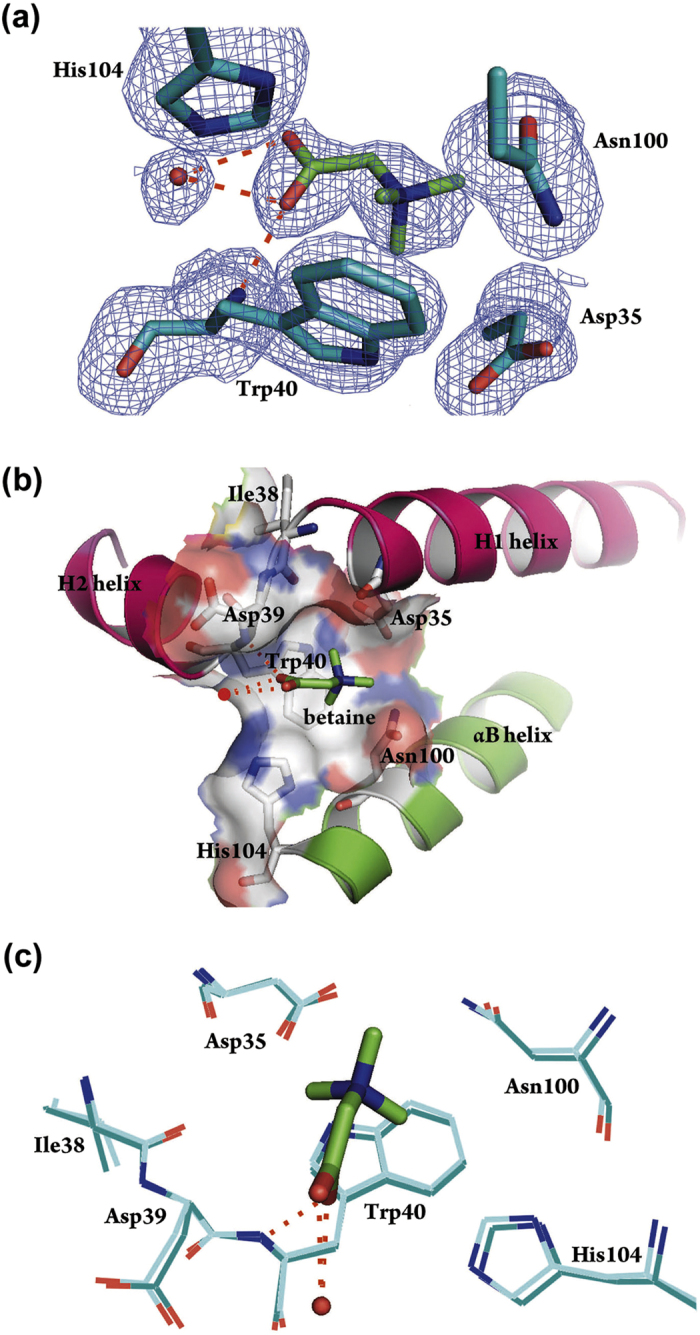
Identification of the betaine-binding site in *Mp*GSMT. (**a**) Electron density map of bound betaine. As shown in the unbiased *F*_*o*_-D*F*_*c*_ map (blue mesh) contoured at 2.0 σ, the positions of all non-hydrogen atoms of betaine are well defined. The bound betaine is stabilized by polar, van der Waals, and cation-π interactions. The distances between the C2 and C3 atoms of betaine and the Cδ2, Cε2, Nε1, and Cδ1 atoms of the indole ring of Trp40 are C2-Nε1: 3.5 Å; C2-Cδ1: 3.6 Å; C3-Cδ2: 3.4 Å; C3-Cε2: 3.5 Å, and the relative orientation between the positively charged quaternary amine and aromatic system fit the criteria for cation-π interaction^52^. (**b**) Electrostatic surface representation of the betaine-binding site. The positively and negatively charged regions are in blue and red, respectively. The helices H1, H2, and αB are shown in cartoon. (**c**) Structural alignment of *Mp*GSMT-betaine (green) with *Mp*GSMT-sarcosine-SAH (cyan) shows that a betaine binding site is present in the inactive form of *Mp*GSMT and that betaine fits snugly in the binding pocket without affecting the conformation of surrounding residues. Hydrogen bonds between the carboxyl group of betaine with the main-chain amide nitrogen of Trp40 and a water molecule (red filled circle) are displayed as red dashed lines. Betaine and selected residues are shown in thick and thin sticks, respectively.

To control the throughput of the betaine biosynthetic pathway, the activity of *Mp*GSMT is known to be feedback inhibited when the end product betaine reaches a sufficiently high concentration^20^,^25^. However, the mechanism by which betaine regulates *Mp*GSMT activity has remained elusive. We speculated that betaine may exert its inhibitory function by preferentially interacting with, and thus stabilizing, the inactivated state of *Mp*GSMT. Given the assumption that the *Mp*GSMT structure determined in this study may represent a functionally inactivated form of the enzyme, we tested whether this structure may possess a betaine binding site by soaking the crystals in a substituted mother liquor containing 1.7 M betaine. The use of such a high concentration of betaine for soaking is physiologically justifiable because upon hyperosmotic shock, betaine-mediated feedback inhibition only engages when its intracellular level approaches approximately 1 M; betaine concentrations as high as 2 M are needed to completely abolish *Mp*GSMT activity^20^,^25^. An unbiased *F*o-*F*c omit map calculated after initial refinement cycles, using a data set collected from a betaine-soaked crystal with just the protein as the source of phases, clearly shows the extra density, which can be readily interpreted as a bound betaine molecule (Fig. 3a). This result demonstrates that the observed *Mp*GSMT structure indeed harbors a pocket for interacting directly with betaine.

The betaine-binding pocket is composed of residues from the H1 helix (Asp35), H1-H2 loop (Ile38, Asp39), H2 helix (Trp40), and αB helix (Asn100, His104) (Fig. 3b). Remarkably, whereas the *Mp*GSMT-sarcosine-SAH structure shows that the carboxyl group of the active site-bound sarcosine is extensively involved in the mediation of the enzyme-substrate interaction (Fig. 2b), no significant interaction was observed between the carboxyl group of betaine and surrounding residues. The bound betaine is mainly stabilized via van der Waals interactions with surrounding residues and the formation of a unique cation-π interaction between the positively charged trimethylated amino group and the indole sidechain of Trp40. A similar charge-aromatic ring interaction was also reported in the structures of betaine transporters^50^,^51^,^52^. The lack of engagement with the carboxyl group suggests that betaine binds only weakly to this pocket and explains why inhibition of *Mp*GSMT activity requires a high concentration of betaine. Although the affinity between betaine and *Mp*GSMT appears weak, the interaction is nevertheless highly specific, as no secondary sites were observed despite the high betaine concentration. The functional significance of the observed betaine-binding pocket is also implicated by the finding that the key interacting residues (Asp35, Trp40, Asn100) are highly conserved among the GSMTs but not among the closely related GNMTs. This suggests that GSMT has evolved to respond to betaine.

Asn100 and His104, two of the residues that constitute the betaine-binding pocket, were mutated to Gln and Lys, respectively, to produce *Mp*GSMT^N100Q^ and *Mp*GSMT^H104K^ for further examining the functional relevance of the observed betaine-binding pocket (Supplementary Fig. S5). Compared to the wild-type enzyme, *Mp*GSMT^H104K^ exhibited increased sensitivity to betaine. It is possible that the longer, positively charged side-chain of Lys enhances betaine-mediated inhibition by interacting with the carboxyl group of betaine. However, *Mp*GSMT^N100Q^ displayed a decreased response to betaine, likely due to the presence of steric clashes between betaine and the longer Gln side-chain. However, the effects of the Asn100 mutation must be interpreted with caution, as this residue appears to serve an important role in cofactor binding by forming hydrogen bounds with the Thr70 of the glycine rich motif. The activity of *Mp*GSMT^N100Q^ is significantly lower that the wild-type enzyme. Nevertheless, it appears that both residues are involved in the betaine-mediated regulation of *Mp*GSMT. Because most betaine-interacting elements are located within the N-terminal region of *Mp*GSMT, it is likely that the movement of this region is restricted upon betaine-binding and traps the enzyme in the inactivated state. The successful identification of a specific betaine-binding site not only supports our hypothesis that the observed *Mp*GSMT structure represents a physiologically relevant, inactivated state of the enzyme but also implicates the structural basis by which betaine inhibits *Mp*GSMT function.

### Structural modeling of *Mp*GSMT in its catalytically active state

To provide structural information for *Mp*GSMT in its catalytically active form, we performed an extensive screen of crystallization conditions in conjunction with different combinations of ligands (substrates, products, and cofactors in various concentrations) to identify the distinct conditions under which *Mp*GSMT crystals can be produced (Supplementary Table S1). However, diffraction analysis revealed that all these *Mp*GSMT crystals were isomorphous with the initial crystals of the *Mp*GSMT-sarcosine-SAH complex (Table 1), with the same space group and unit cell parameters. That is, all the newly determined structures corresponded to the inactivated state. This result suggests that it will be challenging to crystallize *Mp*GSMT in the active conformation for structural analysis, possibly because the inactivated form is relatively stable and abundantly populated in solution. Therefore, the crystal structure of GNMT complexed with acetate and SAM (PDBid: 1NBH^29^), which has been used to elucidate the catalytic mechanism of GNMT and shares significant sequence identity (28.6%) and structural similarity (DALI Z-score 26.2) with *Mp*GSMT, was used as a template to build a homology model for an alternative conformational state of *Mp*GSMT that could correspond to the active form.

**Figure 4:**
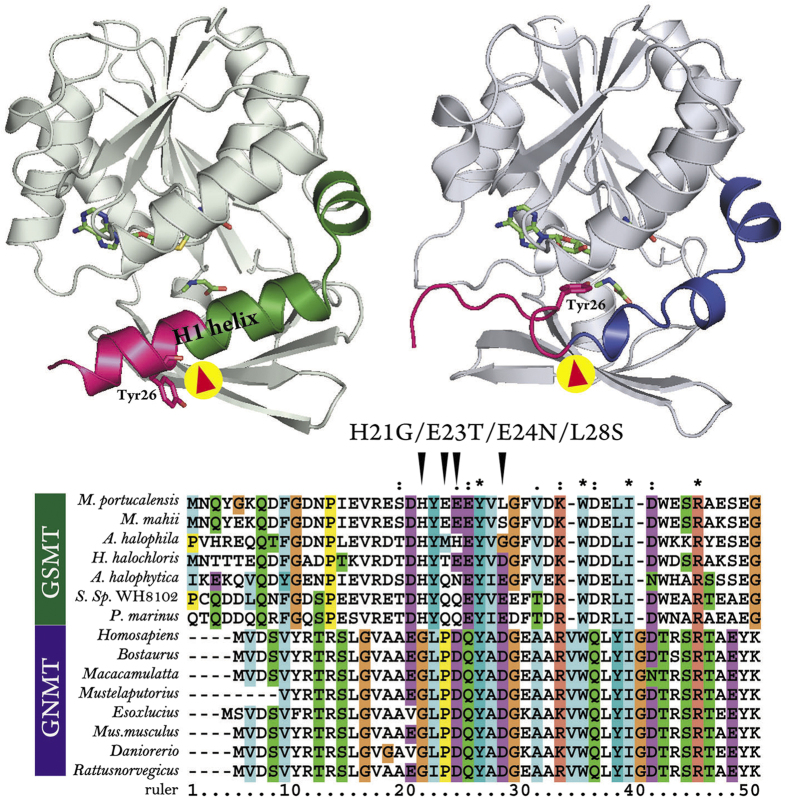
Comparison of *Mp*GSMT-sarcosine-SAH crystal structure and a homology model of *Mp*GSMT constructed using the GNMT-acetate-SAM structure as a template. In the experimentally determined *Mp*GSMT-sarcosine-SAH structure (upper left panel), which adopts a catalytically inactive conformation, the H1 helix is composed of four complete helical turns. In contrast, the corresponding region (red arrowhead) exists as a loop in the modeled, active form of *Mp*GSMT (upper right panel). A key difference between the two structures is that Tyr26 is solvent-exposed in the crystal structure but points towards the cofactor and substrate (green sticks) and becomes an integral part of the active site. The polypeptide segment from His21 to Leu28 (highlighted in scarlet) was chosen for introducing helix-destabilizing mutations. Specifically, residues His21, Glu23, Glu24, and Leu28 were mutated to glycine, threonine, asparagine, and serine, respectively. These amino acids are present in other members of the GSMT family or conserved in the GNMT family (lower panel).

**Figure 5:**
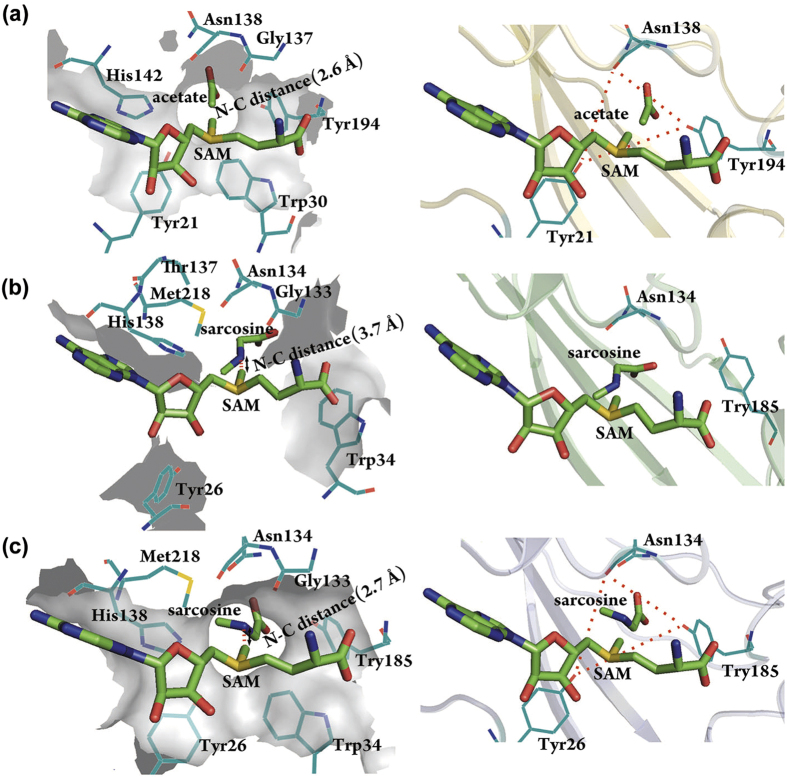
Surface representation showing structural differences of the active site between the two conformational states of *Mp*GSMT. (**a**) In the GNMT structure (PDBid: 1NBH), the estimated N-C distance is approximately 2.6 Å (left panel), and a narrow channel connects the methyl group and the substrate with three electronegative oxygen atoms from Tyr21, N138 and Tyr194 lining the channel wall (right panel). These structural features are expected to facilitate the methyl transfer reaction. (**b**) A 3.7 Å N-C distance was observed in the crystal structure of *Mp*GSMT-sarcosine-SAM (left panel). Because H1 helix, which carries Tyr26 and Trp34, points away from the core domain, only one electronegative group (the main-chain carbonyl group of Asn134) is present in the active site (right panel) and the channel that links the substrate and the methyl group is not present, suggesting that this structure likely reflects a catalytically inactive state for *Mp*GSMT. (**c**) In the *Mp*GSMT structure modeled using GNMT (PDBid: 1NBH; panel (**a**)) as the template, the N-C distance shortens to 2.7 Å (left panel), and a channel lined by three oxygen atoms from Tyr26, N134 and Tyr185 is formed between the methyl group and substrate (right panel). A helix-to-loop transition of the H1 helix allows Tyr26 and Trp34 to enter and complete the assembly of the active site, indicating that the modeled structure may represent *Mp*GSMT in its active conformation.

Guided by the locations of bound acetate and SAM in the GNMT template structure^29^, which occupy the substrate and cofactor binding pocket, respectively, the substrate (either glycine or sarcosine) and SAM were readily docked into the homology model to generate a hypothetical model for the *Mp*GSMT-substrate-cofactor ternary complex (Fig. 4). Compared to the inactive *Mp*GSMT, the H1 helix of N-terminal region undergoes a helix-to-loop transition and moves towards the bound substrate and cofactor. This conformational change brings additional residues into contact with the substrate and cofactor to shield the active site from bulk solvent. Moreover, a channel is assembled between the amino group of the substrate and the methyl group of SAM; the channel walls are lined by triangularly arranged oxygen atoms (the phenolic oxygen atoms of Tyr26 and Tyr185 and the main-chain carbonyl oxygen of Asn134) (Fig. 5). These three electronegative oxygen atoms may facilitate catalysis by defining the pathway of methyl transfer and stabilizing the developing partial positive charges on the three methyl hydrogens during the reaction.

**Figure 6:**
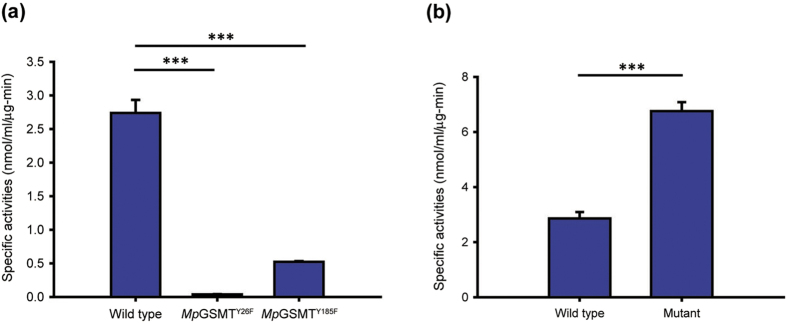
Comparing the specific activities of wild-type and mutant forms of *Mp*GSMT. (**a**) The specific activities of *Mp*GSMT^Y26F^ and *Mp*GSMT^Y185F^ are 74-fold and 5-fold lower than wild-type *Mp*GSMT, respectively. Reactions were performed in a mixture containing 1 mM SAM, 2 M KCl, and 0.2 M glycine. (**b**) The *Mp*GSMT^4mut^ activity is 2.36-fold higher than wild-type *Mp*GSMT under a low-salt (0.1 M KCl) condition containing 1 mM SAM and 0.15 M glycine. All data represent the mean ± standard deviation from three independent experiments. Statistically significant differences are indicated by asterisks (Student’s *t*-test, ***p < 0.001).

Consistent with the proposed catalytic roles of Tyr26 and Tyr185, the Tyr26→Phe and Tyr185→Phe mutant enzymes exhibited 74- and 5-fold reduction in specific activity, respectively (Fig. 6a). The more pronounced functional impact of Tyr26→Phe may be explained by the modeling-based prediction that the phenolic oxygen of Tyr26 is also properly positioned to stabilize the sulfonium group of SAM (Fig. 5c), an interaction known to facilitate methyl transfer reactions^38^,^39^,^40^,^41^. It should also be noted that the distance between the methyl-accepting nitrogen and the methyl carbon is shortened from approximately 3.7 Å in the inactivated state to approximately 2.7 Å in the homology model (Fig. 5). This value is comparable to those seen in the structures of other SAM-dependent N-methyltransferases in their active forms^29^,^43^. Together, these results indicate that the modeled conformational state of *Mp*GSMT indeed harbors the structural features expected for the catalytically active *Mp*GSMT and can be used to successfully predict the role of certain residues in catalysis.

### Disruption of the H1 helix increases *Mp*GSMT activity

To further test whether the functional switching of *Mp*GSMT from the inactivated into active state requires a helix-to-loop transition of the H1 helix of the N-terminal region (Fig. 4), we examined whether reducing the stability of the H1 helix could enhance *Mp*GSMT activity by lowering the threshold of the conformational change. Four residues in the H1 helix (His21, Glu23, Glu24, Lys28) show a high propensity for helix formation and are not directly located in the active site (Figs 2c and 5c). These residues were mutated to amino acids with considerably lower helical propensity (Gly, Thr, Asn and Ser, respectively) to give a tetramutated *Mp*GSMT (*Mp*GSMT^4*mut*^). We chose to use the tetramutant rather than single mutants because simultaneous incorporation of four mutations would be a more effective design to disrupt an α-helix. This approach is considered valid because only a gain-of-function phenotype would allow us to draw a conclusion. It should be noted that none of the four mutation sites are conserved and all four were replaced by residues found in other members of the GSMT family or conserved in the GNMT family. Therefore, the structural integrity of the enzyme should not be significantly affected by our mutant design. Indeed, *Mp*GSMT^4mut^ was abundantly expressed and purified to homogeneity, and the crystal structure of *Mp*GSMT^4mut^ was essentially identical to the wild-type enzyme, except that the N-terminus of H1 helix prior to residue Glu25 underwent a helix-to-loop transition (Supplementary Fig. S6). Consistent with our assumption, the introduction of helix-destabilizing mutations increased the activity by 2.36-fold compared to wild-type *Mp*GSMT (Fig. 6b), suggesting that the presence of the H1 helix may be indicative of the catalytically inactivated enzyme and that enzyme activation can be triggered by a conformational change involving the disruption of this helix.

## Discussion

In this work, we report the crystal structures of *Mp*GSMT, which represents the first structural elucidation of the GSMT family of methyltransferases. Interestingly, despite the simultaneous presence of both substrate (sarcosine) and cofactor (SAH) in the structure, the enzyme appears to adopt a catalytically inactivated conformation. Additional structural changes were required to complete the active site assembly (Fig. 2). The validity of this hypothesis is backed by the finding that crystals of *Mp*GSMT-sarcosine-SAM ternary complex can be prepared without triggering the methyl transfer (Fig. 5b). Given that *Mp*GSMT is subjected to feedback inhibition by betaine^25^, we suspected that the observed structural state of *Mp*GSMT may harbor a betaine-binding site if this structure indeed corresponds to a functionally relevant, inactive form of the enzyme. The successful identification of a betaine-binding pocket composed of residues conserved in the GSMT family provides additional support for our interpretation (Fig. 3). Next, to understand how *Mp*GSMT may switch from an inactive state to an active form, a homology model of activated *Mp*GSMT was constructed (Fig. 4) and validated by mutagenesis (Fig. 6). By comparing the crystal structure of the inactivated state and the experimentally tested homology model of the activated state, we obtained new insights into the structural basis by which regulation of *Mp*GSMT activity is achieved.

First, structural comparison revealed that the activation of *Mp*GSMT likely involves a conformational change in the N-terminal region, because residues 21–37 form an H1 helix in the inactive state. These residues are predicted to undergo a helix-to-loop transition upon activation (Fig. 4). This hypothesis is consistent with the finding that *Mp*GSMT activity can be enhanced when helix-disrupting mutations are introduced into the H1 helix (Fig. 6), which favors conversion to the activated state. Our structural analysis also shows that the H1 helix is an integral part of the betaine-binding pocket (Fig. 3). Thus, the presence of betaine is expected to inhibit catalysis by stabilizing the H1 helix against structural transition. As the betaine-binding pocket is present only in the inactive state, due to structural changes in the H1 helix and rearrangement of the betaine-interacting residues (Fig. 3), we speculate that betaine exerts its inhibitory function by binding to and arresting *Mp*GSMT in the inactive state. An extensive survey of the structures of class I methyltransferases currently available in the Protein Data Bank revealed that the N-terminal regions may exist as a loop, β-strand or α-helix. Although no apparent correlation was observed between the intrinsic methyltransferase activity and the structure adopted by the respective N-terminal region, the observed structure-activity connection of *Mp*GSMT nevertheless suggests that the activity of methyltransferases may be regulated by controlling the threshold of active-site assembly.

Although GNMT and GSMT share a high degree of sequence and structural similarity, a major difference between the two is that GNMT only acts on glycine, whereas GSMT can methylate both glycine and sarcosine. Comparing the GNMT structure with the *Mp*GSMT model revealed that the substrate-binding site of *Mp*GSMT is considerably more spacious (Supplementary Fig. S7). Either glycine or sarcosine can be snugly placed into the *Mp*GSMT active site without inducing steric conflict. The extra methyl group of sarcosine can be accommodated in a side pocket and forms favorable van der Waals interactions with His138 and Met218. In contrast, steric clashes between the methyl group and surrounding residues were observed when sarcosine was modeled in the GNMT active site. Our modeling analysis suggests that differences in shape and volume between the active sites of GNMT and GSMT may explain their distinct substrate specificity. The following similar conclusions have been drawn from structural studies of the histone lysine methyltransferases^53^,^54^: those capable of catalyzing consecutive rounds of methylation, such as human lysine methyltransferase SET7/9 and a viral histone H3 lysine 27 methyltransferase, are known to possess a larger pocket that allows the binding of either mono- or dimethyllysine.

In conclusion, we report here the first crystal structure of a GSMT methyltransferase family member. In addition, we provide structural and functional evidence that the observed conformational state represents *Mp*GSMT in a catalytically inactivated form. Most importantly, we determined the crystal structure of the *Mp*GSMT-betaine binary complex. This structure offers the first visualization of a biosynthetic methyltransferase in complex with a *bona fide* feedback inhibitor, that is, the end product of the corresponding biosynthesis pathway. Taken together, this work reveals structural insights into GSMT function and suggests a structural basis by which feedback inhibition of biosynthetic methyltransferases may be achieved.

## Methods

### Protein preparation

The construction of an expression plasmid for producing full-length *Mp*GSMT (residues 1–263) with a N-terminal His-tag was described previously^25^. QuikChange^TM^ mutagenesis method (Stratagene) was applied to this vector to allow the production of two *Mp*GSMT mutants: *Mp*GSMT^Y26F^ and *Mp*GSMT^Y185F^. The wild-type and mutant proteins were produced in *E. coli* strain BL21-CodonPlus(DE3)-RIL(Cam^r^). Briefly, single colonies were inoculated into LB medium containing 50 μg/ml kanamycin and 34 μg/ml chloramphenicol, cells were grown at 37 °C until OD_600_ reached ~0.6. Protein expression was induced by adding isopropyl-β-D-thiogalactopyranoside to a final concentration of 1.0 mM, and culture was shifted to 30 °C and grown for another 20 hours. The cells were harvested by centrifugation and stored at −80 °C until further use. For protein purification, the cell pellet from 1 liter of culture was resuspended in 10 ml of buffer A (50 mM Tris-HCl pH 7.5, 300 mM KCl, 0.5 mM PMSF, 1 mM β-mercaptoethanol). Following cell disruption by sonication, the crude cell lysate was clarified by centrifugation at 27,216 × g for 1 hour (repeat 3 times) at 4 °C and loaded onto a pre-equilibrated Ni-NTA column. The column was washed with ten column volumes of buffer A and a step-wise increasing of imidazole concentration from 15 mM to 75 mM were used to remove contaminating proteins. The bound *Mp*GSMT was eluted using buffers containing 150 mM and 300 mM imidazole. Fractions containing *Mp*GSMT were pooled and concentrated using an Amicon Ultra device (Millipore; 30 KDa cutoff) and then loaded on to a 16/60 Superdex-200 size-exclusion column (GE Healthcare) pre-equilibrated in gel-filtration buffer (100 mM TES pH 7.3, 2.0 M KCl, 1.0 mM EDTA, 1.0 mM β-Mercaptoethanol). The peak fractions containing *Mp*GSMT were pooled and concentrated by ultrafiltration to 6.6 mg/ml for crystallization.

### Protein crystallization, Data collection and structural determination

Initial crystallization trials were performed with commercially available kits (Hampton Research) using the hanging-drop vapor-diffusion method. To obtain the crystals of *Mp*GSMT-sarcosine-SAH ternary complex, purified *Mp*GSMT (6.6 mg/ml) was first incubated with 0.1 mM SAH and 0.2 M sarcosine on ice overnight, followed by mixing 1.0 μl of the protein sample with 1.0 μl of reservoir solution containing 0.1 M Tris-HCl pH 8.0, 1.5 M sodium chloride and equilibrated at 4 °C against 200 μl of reservoir solution. The crystals can be obtained in ~3–4 weeks. Before freezing, the crystals were transferred into 20 μl of substitute mother liquor containing 0.1 M TES pH 8.0, 1.8 M NaCl, 0.51 M sarcosine, 0.1 mM SAH and 25% ethylene glycol for 48 hours, and flash-frozen by plunging into liquid nitrogen. Crystals of the *Mp*GSMT-SAM binary complex were prepared by transferring the *Mp*GSMT-sarcosine-SAH ternary complex crystals into a substitute mother liquor 0.2 M potassium sodium tartrate tetrahydrate, 23% (w/v) polyethylene glycol 3350, 2 mM SAM and 25% ethylene glycol for 48 hours. Crystals of the *Mp*GSMT-sarcosine-SAM ternary complex were prepared by transferring the *Mp*GSMT-SAM binary complex crystals into a substitute mother liquor 0.2 M potassium sodium tartrate tetrahydrate, 23% (w/v) polyethylene glycol 3350, 0.685 M sarcosine, 2 mM SAM and 25% ethylene glycol for 48 hours. Crystals of the both *Mp*GSMT-SAM and *Mp*GSMT-sarcosine SAM were looped directly from the respective substitute mother liquor and harvested by plunging into liquid nitrogen. To obtain the crystals of betaine-bound *Mp*GSMT, 1.0 μl of purified *Mp*GSMT (6.6 mg/ml) was mixed with 1.0 μl of reservoir solution containing 0.2 M potassium sodium tartrate tetrahydrate, 20% (w/v) polyethylene glycol 3,350 and equilibrated at 4 °C against 200 μl of reservoir solution. Single crystals can be obtained in ~3–4 weeks. Before freezing, 1.0 μl of 5.0 M betaine was added into the crystallization drop and incubated for ~3 hours at 4 °C, and the betaine-soaked crystals were frozen in liquid nitrogen using Parabar 10312 (Hampton research) as the cryoprotectant. Specifically, theses crystals were transferred to a drop of Parabar 10312 and the residual solvent which surrounded the crystal was removed with the CryoLoop, flash-frozen was achieved by plunging into liquid nitrogen. To obtain the crystals of *Mp*GSMT^4Mut^ (H21G,E23T,E24N,L28S), purified *Mp*GSMT^4Mut^ (6.6 mg/ml) was first incubated with 0.1 mM SAH and 50 mM glycine on ice overnight, followed by mixing 1.0 μl of the protein sample with 1.0 μl of reservoir solution containing 0.2 M ammonia phosphate monobasic. Before freezing, the crystals were transferred into 20 μl of substitute mother liquor containing 0.23 M ammonia phosphate monobasic, 0.1 mM SAH and 3.35 M betaine as the cryoprotectant for 24 hours, and flash-frozen by plunging into liquid nitrogen.

X-ray diffraction data sets were collected at NSRRC beamlines BL15A1 and BL13C1 (Hsinchu, Taiwan) and SPring8 beamline BL12B2 (Japan) and processed using the HKL2000 program suit^55^. The *Mp*GSMT-sarcosine-SAH was solved by molecular replacement using the rat GNMT structure (PDBid: 1XVA) as the search model. The MR-phased initial structure model was improved by the AutoBuild module of Phenix^56^ followed by rounds of manual adjustment and refinement using Coot^57^ and Phenix. The *Mp*GSMT-sarcosine-SAH structure was then used as the search model to solve the *Mp*GSMT-SAM, *Mp*GSMT-sarcosine-SAM, *Mp*GSMT-betaine and *Mp*GSMT^4Mut (H21G, E23T, E24N, L28S)^ structures. In all cases, the initial m*F*_o_-D*F*_c_ difference electron density maps allow the bound ligands (sarcosine, SAH, SAM, betaine) to be located and precisely positioned using Coot. The structures then underwent rounds of manual model rebuilding and refinement using Coot and Phenix. Data collection and refinement statistics were summarized in Table 1. All figures were generated using PyMOL^58^.

### Homology modeling of the active form of *Mp*GSMT

To identify a suitable template for modeling, the crystal structure of *Mp*GSMT reported in this study was used for a DALI structural similarity search. The structure of rat liver GNMT in complex with the cofactor (SAM) and inhibitor (acetate) (PDBid: 1NBH, chain D) was chosen for the extensive similarity between the corresponding catalytic cores and lid domains, and because the active site of GNMT is in a catalytically active conformation^29^. Sequence alignment and homology modeling were performed by Accelrys Discovery Studio (Accelrys Software Inc.). SAM and acetate were placed into the homology model of *Mp*GSMT by transferring the atomic coordinates of the ligands after superimposing GNMT-acetate-SAM complex onto the homology model.

### Introducing mutations that destabilize helix H1 into *Mp*GSMT

The H1-destabilizing mutations (H21G/E23T/E24N/L28S) were introduced into the *Mp*GSMT gene by SOE (gene Splicing by Overlap Extension) method^59^. The primers used for mutagenesis were synthesized by the Viogene BioTek Corp, Taiwan (Supplementary Table S2). PCR reactions were performed using the wild-type *Mp*GSMT gene as the template and either the primer pair a/d or b/c to obtain the 5′ and 3′ fragments of the gene, respectively. The two PCR-amplified products were retrieved by gel extraction and mixed, the mixture then underwent one cycle of primer-free extension to generate small amount of full-length *Mp*GSMT gene that carries the L28S mutation, which served as the template for subsequent PCR amplification cycles using primer pair a/b to produce large amount of the mutated *Mp*GSMT gene. In the next round of SOE, PCR reactions were performed using the mutated *Mp*GSMT gene as the template and either the primer pair a/f or b/e to obtain the 5′ and 3′ fragments of the gene, respectively. Similarly, the new PCR-amplified fragments were gel extracted, mixed, and underwent primer-free extension, followed by additional PCR cycles in the presence of the primer pair a/b to produce full-length H21G/E23T/E24N/L28S mutated *Mp*GSMT gene. The mutated *Mp*GSMT genes were inserted into pET28a for sequencing and protein production.

### Activity assay

The “SAM510: SAM Methyltransferase Assay Kit” (G-Biosciences) was used for measuring the activities of wild-type and mutant *Mp*GSMT. The assays were set up and performed in a 96-well plate according to the manufacturer’s suggestion. Each reaction mixture (143.5 μL) contains 7 μg of enzyme, 150 mM glycine, 1 mM SAM, 0.1 M KCl, and 100.5 μL of the master mix. The reaction was followed using a microplate spectrophotometer (PowerWave, BioTek Instruments, Inc., USA) to monitor the absorbance change at 510 nm under 37 °C. Absorbance of the reaction mixture was recorded every minute for a total of 1 hour. A radioactivity-based assay described previously was used to measure the activities of *Mp*GSMT^Y26F^ and *Mp*GSMT^Y185^ 25.

### Statistical analysis

Data obtained in this study were examined with Student’s *t*-test embedded in SigmaPlot. Confidence levels of Student’s *t*-tests were depicted in the graph, three asterisks indicating *p* < 0.001.

## Additional Information

**Accession codes:** The crystal structures of *Mp*GSMT-sarcosine-SAH, *Mp*GSMT-SAM, *Mp*GSMT-sarcosine-SAM, *Mp*GSMT-betaine, *Mp*GSMT^4Mut^(H21G, E23T, E24N, L28S) have been deposited in wwPDB under accession codes 5HIL, 5HIK, 5GWX, 5HIJ, and 5H02, respectively.

**How to cite this article**: Lee, Y.-R. *et al*. Structural Analysis of Glycine Sarcosine N-methyltransferase from *Methanohalophilus portucalensis* Reveals Mechanistic Insights into the Regulation of Methyltransferase Activity. *Sci. Rep.* **6**, 38071; doi: 10.1038/srep38071 (2016).

**Publisher's note:** Springer Nature remains neutral with regard to jurisdictional claims in published maps and institutional affiliations.

## Supplementary Material

Supplementary Information

## Figures and Tables

**Table 1:** Summary of crystallographic analysis.

Crystal structure	*Mp*GSMT-sarcosine-SAH	*Mp*GSMT-SAM	*Mp*GSMT-sarcosine-SAM	*Mp*GSMT-betaine	*Mp*GSMT^4Mut^ (H21G,E23T,E24N,L28S)
PDBid	5HIL	5HIK	5GWX	5HIJ	5H02
Space group	*I*222	*I*222	*I*222	*I*222	*I*222
Unit cell dimensions					
* a, b, c* (Å)	51.9, 121.1, 131.6	51.9, 121.0, 131.8	51.7, 120.7, 130.9	52.2, 120.5, 131.4	51.9, 120.8, 131.3
Data collection					
* *Wavelength (Å)	0.97622	0.97622	0.97622	1.00000	1.00000
* *Resolution range (Å)	30.0–2.47 (2.60–2.47)	30.0–2.35 (2.43–2.35)	50.0–2.20 (2.28–2.20)	20.0–1.94 (2.0–1.93)	20.0–1.78 (1.82–1.78)
* *Observed reflections	51428	80639	72957	133148	196668
* *Unique reflections	15131	17545	20701	31210	40112
* *Completeness (%)	99.0 (100)	99.9 (100)	98.3 (98.4)	99.8 (100.0)	100.0 (100.0)
* *Multiplicity	3.4 (3.4)	4.6 (4.8)	3.5 (3.6)	4.3 (4.3)	4.9 (4.9)
* *Mean I/sigma (I)	13.6 (2.6)	22.8 (4.9)	18.1 (2.7)	21.7 (3.3)	31.5 (3.4)
* R*_sym_ (%)^a^	8.6 (49.5)	6.3 (38.5)	5.7 (40.0)	5.4 (47.7)	4.4 (49.8)
Refinement					
* *Resolution range (Å)	24.92–2.47 (2.66–2.47)	27.49–2.35 (2.50–2.35)	29.20–2.21 (2.32–2.21)	19.73–1.93 (1.99–1.93)	19.89–1.78 (1.82–1.78)
No. of reflection in					
* *Working set	15129	17543	20701	31208	40110
* *Test set	762	877	1039	1571	1995
* *Wilson B-factor	38.1	37.1	36.0	27.3	24.0
* R*_crys_ (%)^b^	17 (22)	17 (19)	17 (21)	17 (22)	18 (22)
* R*_free_ (%)^b^	22 (29)	21 (26)	20 (25)	21 (27)	20 (22)
Number of atoms	2046	2078	2130	2160	2227
* *Macromolecules	1935	1962	1984	1962	2012
* *Ligands	52	43	49	8	42
* *Waters	59	73	97	190	173
* *No of residues/Total residues	240/263	240/263	249/263	240/263	252/263
* *Missing residues	23	23	14	23	11
r.m.s. deviation from ideal					
* *Bond lengths (Å)	0.008	0.007	0.007	0.007	0.006
* *Bond angles (degrees)	1.04	1.04	0.82	0.96	0.80
Ramachandran analysis^c^					
* *Favored (%)	97.0	96.6	98.0	97.9	98.0
* *Outliers (%)	0	0	0	0	0
Clashscore	1.83	2.33	1.03	1.31	1.78
Average B-factor	45.4	43.9	42.6	32.3	35.6
* *Macromolecules	45.4	43.8	41.7	31.5	32.1
* *Solvent	41.8	42.9	44.7	39.7	40.5

Statistics for the highest-resolution shell are shown in parentheses.

^a^*R*_sym_ = (Σ|*I*_*hkl*_ − <*I*>|)/(Σ*I*_*hkl*_), where the average intensity <*I*> is taken over all symmetry equivalent measurements, and *I*_*hkl*_is the measured intensity for any given reflection.

^b^*R*_cryst_ = (∑∥*F*_*o*_|− *k*|*F*_*c*_∥)/(∑ |*F_o_*|). *R*_free_ = *R*_cryst_ for a randomly selected subset (5%) of the data that were not used for minimization of the crystallographic residual.

^c^Categories were defined by PHENIX^56^.
