# Supplementary material for: Structural Analysis of Glycine Sarcosine N-methyltransferase from Methanohalophilus portucalensis Reveals Mechanistic Insights into the Regulation of Methyltransferase Activity
Source: Sci Rep. 2016 Dec 9;6:38071. doi: 10.1038/srep38071 (PMC5146677; doi:10.1038/srep38071)
Supplement: Supplementary Information [file srep38071-s1.pdf]

# Supplementary Information for

# Structural Analysis of Glycine Sarcosine N-methyltransferase from *Methanohalophilus portucalensis* Reveals Mechanistic Insights into the Regulation of Methyltransferase Activity

**Yi-Ru Lee[1], Te-Sheng Lin[1], Shu-Jung Lai[2], Mu-Sen Liu[1], Mei-Chin Lai[2,*], and Nei-Li Chan[1,3,*]**

**Contents:**

**Supplementary Tables: 1 and 2**
**Supplementary Figures: 1 ~ 7**
**Supplementary References**

# Supplementary Tables

## Supplementary Table S1

**Table S1 Crystallization conditions of *Mp*GSMT**

| Crystallization conditions | buffer | salt | precipitant |
|---|---|---|---|
| 1 | 0.1 M CAPS/sodium hydroxide pH 10.5 | | 30% (v/v) PEG 400 |
| 2 | 0.1 M sodium acetate/ acetic acid pH 4.5 | | 0.8 M sodium phosphate monobasic/ 1.2 M potassium phosphate dibasic |
| 3 | 0.1 M sodium citrate tribasic dihydrate pH 5.6 | 0.2 M ammonium acetate | 30% v/v (+/-)-2-methyl-2,4-pentanediol |
| 4 | | | 25% v/v ethylene glycol |
| 5 | 0.1 M Tris HCl pH 8.5 | | 1.0 M ammonium Sulfate |
| 6 | 0.1 M sodium cacodylate pH 6.5 | 0.2 M magnesium acetate | 15% v/v (+/-)-2-methyl-2,4-pentanediol |
| 7 | 0.1 M sodium citrate pH 5.6 | 0.2 M ammonium acetate | 15% v/v (+/-)-2-methyl-2,4-pentanediol |
| 8 | 0.05 M sodium cacodylate trihydrate pH 6.0 | 0.04 M magnesium acetate tetrahydrate | 30% v/v (+/-)-2-methyl-2,4-pentanediol |
| 9 | | 0.2 M magnesium acetate tetrahydrate | 20% w/v polyethylene glycol 3,350 |
| 10 | | 0.2 M sodium acetate trihydrate | 20% w/v polyethylene glycol 3,350 |
| 11 | | 0.2 M calcium acetate hydrate | 20% w/v polyethylene glycol 3,350 |
| 12 | 0.1 M MOPS pH 7.0 | 0.1 M Magnesium chloride | 20% w/v polyethylene glycol 3,350 |
| 13 | 0.1 M HEPES pH 7.5 | | 12% w/v polyethylene glycol 3,350 |
| 14 | 0.1 M HEPES pH 7.5 | | 5% v/v (+/-)-2-Methyl-2,4-pentanediol<br>10% w/v polyethylene glycol 10,000 |
| 15 | 0.1 M HEPES pH 7.5 | | 3.0 M sodium chloride |
| 16 | 0.1 M Tris pH 8.5 | | 3.0 M sodium chloride |
| 17 | 0.1 M Bis-Tris pH 6.5 | | 0.5 M magnesium formate dihydrate |
| 18 | 0.1 M HEPES pH 7.5 | | 0.5 M magnesium formate dihydrate |
| 19 | 0.1 M Bis-Tris pH 6.5 | 0.2 M ammonium acetate | 45% v/v (+/-)-2-methyl-2,4-pentanediol |
| 20 | 0.1 M Tris pH 8.5 | 0.2 M sodium chloride | 25% w/v polyethylene glycol 3,350 |
| 21 | | 0.2 M potassium sodium tartrate tetrahydrate | 20% w/v polyethylene glycol 3,350 |

# Supplementary Table S2

**Table S2 Primers for SOE mutagenesis**

| primer | sequence (5' to 3') |
|---|---|
| (a) forward primer (5' primer) | GGACCCATATGAACCAATACGGAAAACAGGATTTTGG |
| (b) reverse primer (3' primer) | GGACCCTCGAGTTAATTTTTTTCTGCAACATGGAGGAAGAAATCAGG |
| (c) forward primer with L28S mutation site | GGTTATACGAACGAGTATGTTAGTGGATTTGTTGATAAATGGGACG |
| (d) reverse primer with L28S mutation site | CGTCCCATTTATCAACAAATCCACTAACATACTCGTTCGTATAACC |
| (e) forward primer with H21G/E23T/E24N mutation sites | GGGAATCGGACGGTTATACGAACGAGTATGTTAGTGG |
| (f) reverse primer with H21G/E23T/E24N mutation sites | CCACTAACATACTCGTTCGTATAACCGTCCGATTCCC |

**Supplementary Figures**

## Supplementary Figure S1

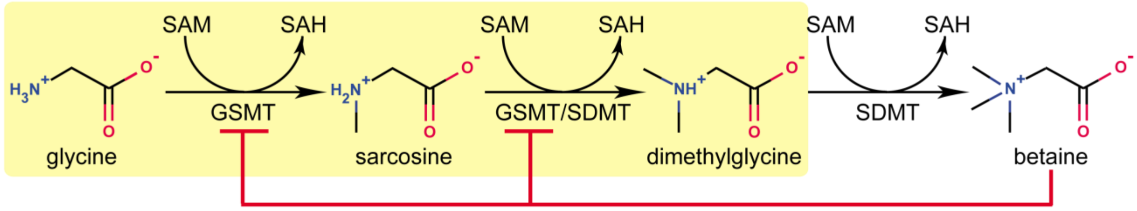


**Figure S1. The *de novo* betaine biosynthesis pathway in *M. portucalensis*.** The glycine-derived betaine is produced in this pathway by a three-step methylation process involving two SAM-dependent methyltransferases: the glycine sarcosine methyltransferase (GSMT) and sarcosine dimethylglycine methyltransferase (SDMT)[1,2]. Glycine is the initial substrate whose amino group undergoes three methylation events to form betaine. GSMT catalyzes the transfer of the first and second methyl groups to form sarcosine and dimethylglycine. The second and third methyl transfer is performed by SDMT to form dimethylglycine and the final product betaine. Both GSMT and SDMT are capable of transforming sarcosine to dimethylglycine. The methylation activity of *Mp*GSMT is known to be negatively regulated by the end product betaine[3].

## Supplementary Figure S2

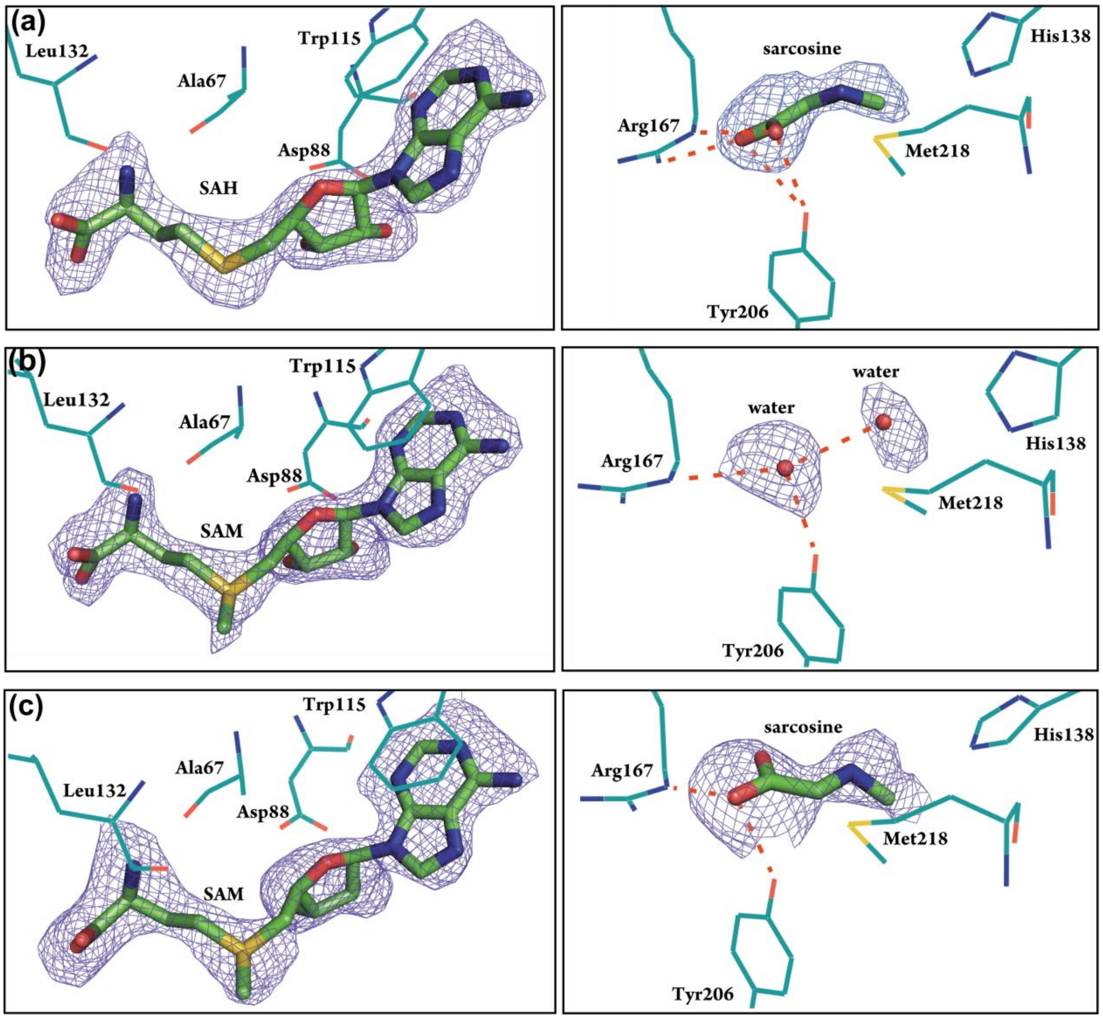


**Figure S2. The final m*Fo*-D*Fc* electron density maps of the bound ligands.** (a) The electron density maps of SAH (left) and sarcosine (right) in the *Mp*GSMT-sarcosine-SAH ternary complex structure. (b) The electron density map of SAM (left) and the bound water molecules (right) in the *Mp*GSMT-SAM binary complex structure. (c) The electron density map of SAM (left) and sarcosine (right) in the *Mp*GSMT-sarcosine-SAM ternary complex structure. All maps were contoured at around 3.0 σ.

**Supplementary Figure S3**

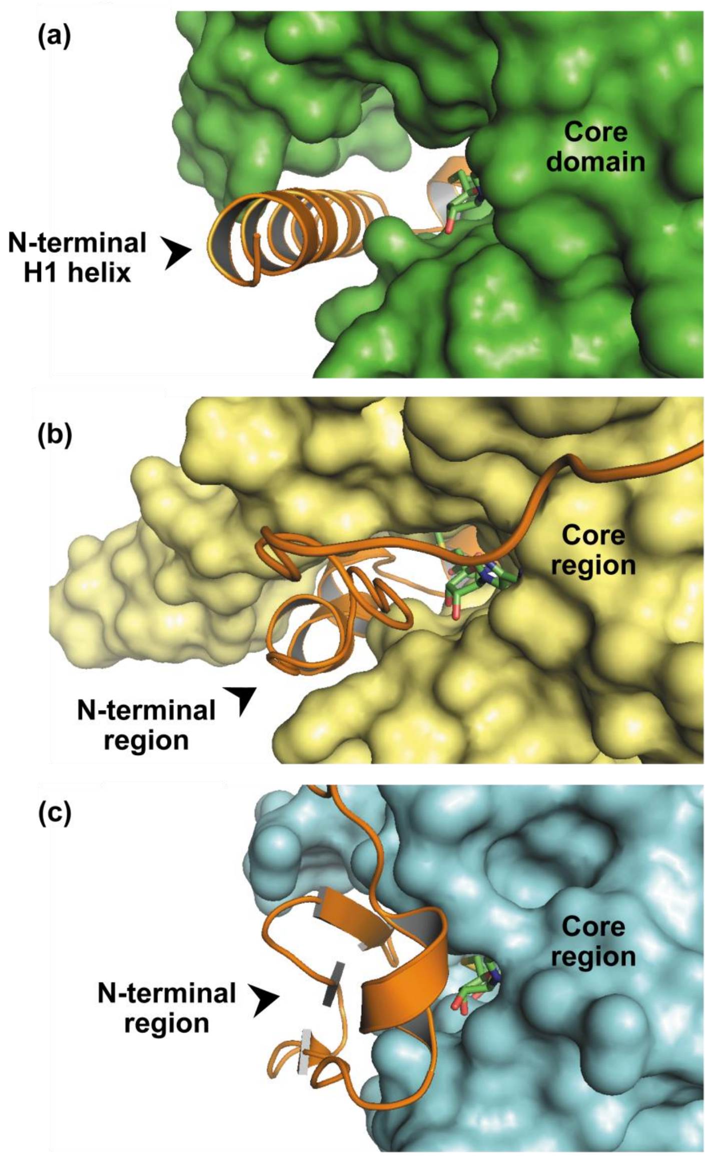


**Figure S3. Surface representations showing the conformational states of the active site of *Mp*GSMT and GNMT.** (a) The active site seen in the *Mp*GSMT-sarcosine-SAH structure adopts a partially open conformation with its N-terminal H1 helix points away from the core domain. In contrast, the active sites of other small molecule methyltransferases, such as GNMT (PDBid: 1NBH; (b))[4] and GAMT (PDBid: 1XCJ; (c))[5] are in closed states. The SAH and SAM are in green sticks, the N-terminal regions are displayed in cartoon representations and colored in orange.

## Supplementary Figure S4

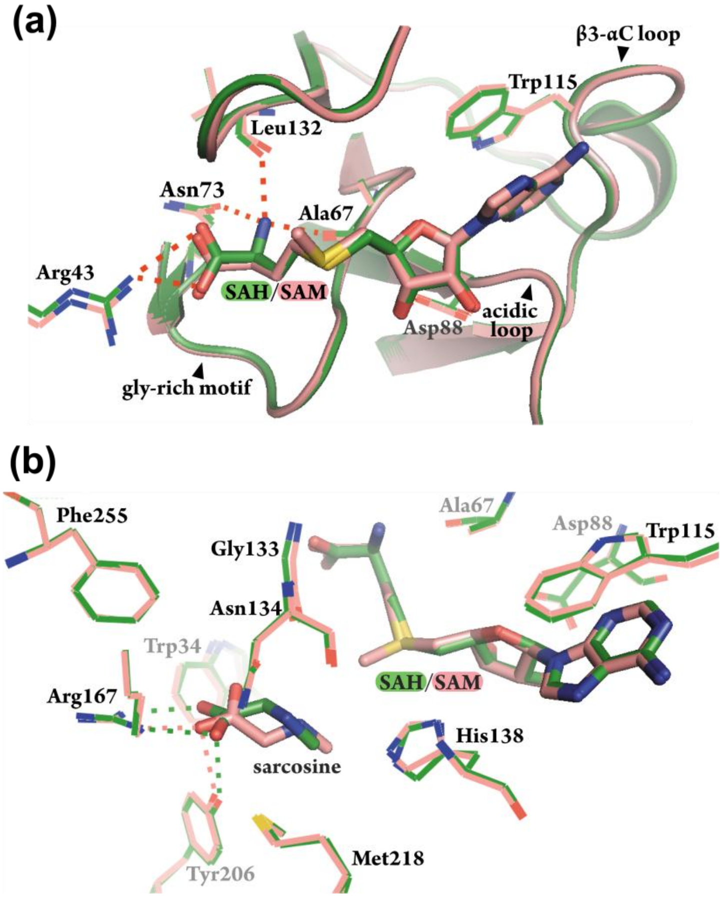


**Figure S4. Structural alignment of *Mp*GSMT-sarcosine-SAH and *Mp*GSMT-sarcosine-SAM.** Superimposition of the cofactor binding pockets of *Mp*GSMT-sarcosine-SAH (green) and *Mp*GSMT-sarcosine-SAM (salmon) revealed that the two structures, along with the bound cofactors, can be perfectly aligned, except for the presence of an extra sulfonium-linked methyl group in SAM (a). Similarly, except for the minor reposition of sarcosine, no significant changes were observed for residues involved in substrate binding (b). The three regions (glycine-rich motif, acidic loop, and loop region between β3 and αC) and residues involved in cofactor/substrate binding are indicated. Hydrogen bonds and salt bridges between ligand/substrate and protein residues are displayed with dashed lines.

**Supplementary Figure S5**

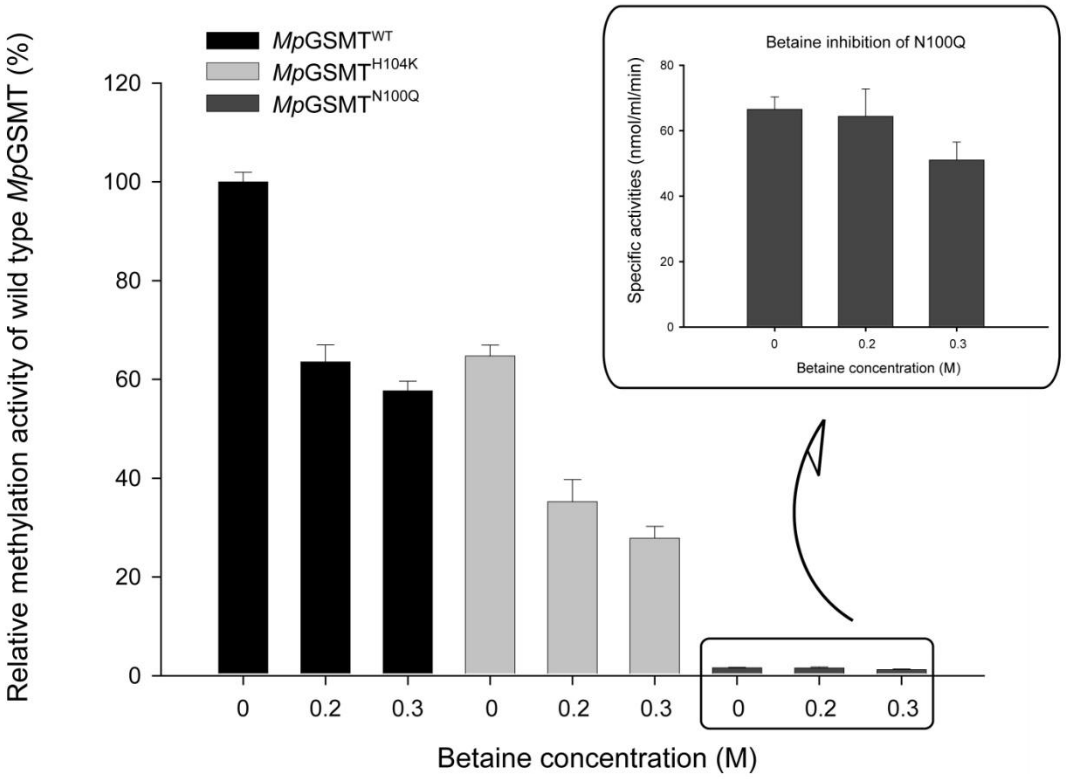


| | *Mp*GSMT$^{WT}$ | | | *Mp*GSMT$^{H104K}$ | | | *Mp*GSMT$^{N100Q}$ | | |
|---|---|---|---|---|---|---|---|---|---|
| Betaine concentration | 0 M | 0.2 M | 0.3 M | 0 M | 0.2 M | 0.3 M | 0 M | 0.2 M | 0.3 M |
| Inhibition (%) per each untreated | 0 | 36 | 42 | 0 | 40 | 52 | 0 | 3 | 23 |

**Figure S5. Effect of betaine-binding site mutants on the betaine-mediated inhibition of *Mp*GSMT activity.** The methyltransferase activities of the wild-type, *Mp*GSMT$^{H104K}$ and *Mp*GSMT$^{N100Q}$ were examined with increasing concentration of betaine. The raw activity data were shown as percentiles using the betaine-free wild-type *Mp*GSMT activity as the reference. To compare the sensitivity of wild-type and mutant *Mp*GSMT to betaine, the activity displayed by each variant in the absence of betaine was used as the reference. Due to the significant impact of the N100Q mutation on enzyme activity, the raw activity data for *Mp*GSMT$^{N100Q}$ is shown in the inset for clarity. All data represents the mean ± standard deviation from three independent experiments.

## Supplementary Figure S6

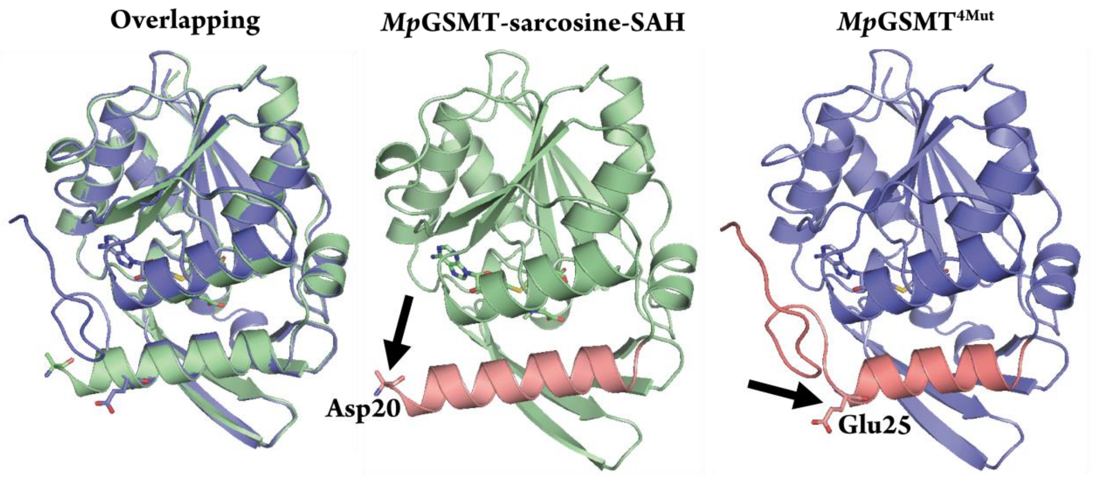


**Figure S6. Structural comparison between *Mp*GSMT-sarcosine-SAH and *Mp*GSMT$^{4Mut}$ revealed a mutation-induced helix-to-loop transition at the N-terminal region of H1 helix.** The N-terminal segment of *Mp*GSMT-sarcosine-SAH (green, middle panel) and *Mp*GSMT$^{4Mut}$ (purple, right panel) are shown in red. While this segment adopts a helical conformation in *Mp*GSMT-sarcosine-SAH, a portion of this segment appears to undergo a helix-to-loop transition in *Mp*GSMT$^{4Mut}$. Except for this change, no significant changes were detected between the two structures (left panel).

**Supplementary Figure S7**

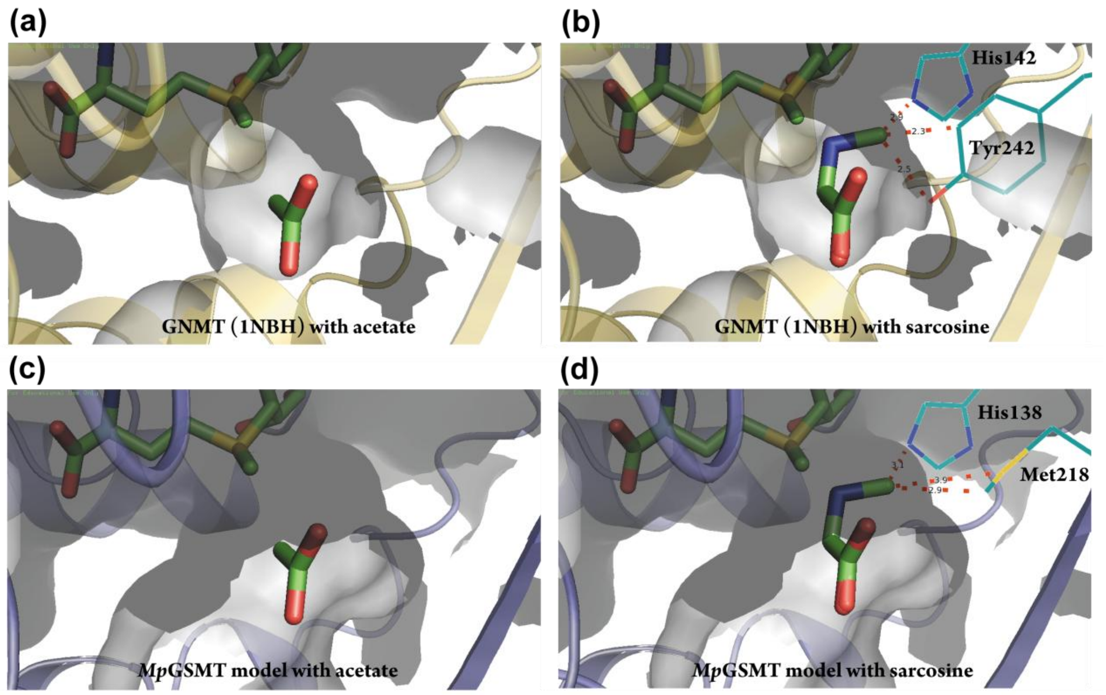


**Figure S7. Structural basis underlying the diverged substrate selectivity between GNMT and *Mp*GSMT**. Surface representations of the substrate-binding pocket of GNMT (PDBid: 1NBH; (a)) and the homology model of *Mp*GSMT (c) in the presence of SAM and acetate. Sarcosine can be readily modeled into the substrate-binding sites of both structures by superposing the carboxyl moiety onto the acetate (b, d). The distances between the sarcosine's N-methyl carbon and adjacent residues are 2.3, 2.5 and 2.9 Å in GNMT (b), and 2.9, 3.1 and 3.9 Å in *Mp*GSMT model (d), indicating that the substrate-binding pocket of GNMT is smaller than *Mp*GSMT and thus cannot accommodate sarcosine.

**Supplementary References**


1 Lai, M. C., Yang, D. R. & Chuang, M. J. Regulatory factors associated with synthesis of the osmolyte glycine betaine in the halophilic methanoarchaeon Methanohalophilus portucalensis. *Applied and environmental microbiology* **65**, 828-833 (1999).

2 Lai, M. C., Wang, C. C., Chuang, M. J., Wu, Y. C. & Lee, Y. C. Effects of substrate and potassium on the betaine-synthesizing enzyme glycine sarcosine dimethylglycine N-methyltransferase from a halophilic methanoarchaeon Methanohalophilus portucalensis. *Research in microbiology* **157**, 948-955, doi:10.1016/j.resmic.2006.08.007 (2006).

3 Lai, S. J. & Lai, M. C. Characterization and regulation of the osmolyte betaine synthesizing enzymes GSMT and SDMT from halophilic methanogen Methanohalophilus portucalensis. *PloS one* **6**, e25090, doi:10.1371/journal.pone.0025090 (2011).

4 Takata, Y. *et al.* Catalytic mechanism of glycine N-methyltransferase. *Biochemistry* **42**, 8394-8402, doi:10.1021/bi034245a (2003).

5 Komoto, J. *et al.* Catalytic mechanism of guanidinoacetate methyltransferase: crystal structures of guanidinoacetate methyltransferase ternary complexes. *Biochemistry* **43**, 14385-14394, doi:10.1021/bi0486785 (2004).
